# On the equivalence between squeezing and entanglement potential for two-mode Gaussian states

**DOI:** 10.1038/s41598-023-38572-1

**Published:** 2023-07-20

**Authors:** Bohan Li, Aritra Das, Spyros Tserkis, Prineha Narang, Ping Koy Lam, Syed M. Assad

**Affiliations:** 1 Department of Quantum Science and Technology, Centre for Quantum Computation and Communication Technology, Australian National University, Canberra, ACT 2601 Australia; 2 Physical Sciences, College of Letters and Science, University of California (UCLA), Los Angeles, CA USA; 3 School of Physical and Mathematical Sciences, Nanyang Technological University, Singapore, 639673 Republic of Singapore; 4 Institute of Materials Research and Engineering, Agency for Science, Technology and Research (A*STAR), Singapore, 138634 Republic of Singapore

**Keywords:** Mathematics and computing, Optics and photonics, Physics

## Abstract

The maximum amount of entanglement achievable under passive transformations by continuous-variable states is called the entanglement potential. Recent work has demonstrated that the entanglement potential is upper-bounded by a simple function of the squeezing of formation, and that certain classes of two-mode Gaussian states can indeed saturate this bound, though saturability in the general case remains an open problem. In this study, we introduce a larger class of states that we prove saturates the bound, and we conjecture that all two-mode Gaussian states can be passively transformed into this class, meaning that for all two-mode Gaussian states, entanglement potential is equivalent to squeezing of formation. We provide an explicit algorithm for the passive transformations and perform extensive numerical testing of our claim, which seeks to unite the resource theories of two characteristic quantum properties of continuous-variable systems.

## Introduction

Entanglement is a non-classical property that can be considered as a resource for various quantum technology applications^1^. In continuous-variable (CV) systems^2^, e.g., systems consisting of bosonic modes, entanglement is connected with a more fundamental property called squeezing^3^. Squeezing constitutes a necessary condition for entanglement in CV Gaussian systems^4–6^ and finds applications in numerous areas of quantum optics and CV quantum information, including metrology^7–9^, secure quantum communication^10–13^, quantum teleportation^14–16^, cluster states^17,18^, heralded gates^19^ and quantum computation^20,21^.

Moreover, any multi-mode squeezed state can be transformed into an entangled state under passive operations^22,23^. Passive operations in CV systems are relatively easier to perform in the laboratory than active operations. There exist multi-mode quantum states that are not entangled, but have the potential to be entangled by simply mixing on a beam splitter^24,25^. Motivated by this, we study the entanglement potential of Gaussian states. Conceptually, the entanglement potential measures the maximum amount of entanglement obtainable under passive operations^24^. This potential depends on the way that entanglement is measured, e.g., in Ref.^24^, logarithmic negativity^26^ was selected for this purpose, whereas in Ref.^25^, the entanglement of formation^27^ was chosen.

Focusing on the entanglement of formation, some of us have previously derived analytic expressions for the entanglement potential of a few specific classes of two-mode Gaussian states: symmetric states and balanced correlated states^25^. These analytic expressions were shown to be directly connected to the squeezing of formation^28^—a measure that quantifies the amount of squeezing in a quantum state. In Ref.^25^, an explicit derivation of the passive operations needed to achieve this potential was provided. Further, it was shown that for general two-mode Gaussian states, a monotonic function, $h_0(\cdot )$, of the squeezing of formation upper-bounds the entanglement potential.

In this work, we extend that analysis in two ways: first, we analytically show that for a larger, six-parameter class of two-mode Gaussian states, the entanglement potential is equal to $h_0(\cdot )$ of the squeezing of formation. Henceforth, we shall refer to all states having entanglement equal to entanglement potential as *potential-saturating* states. Second, we conjecture that any two-mode Gaussian state can be passively transformed into a potential-saturating state from the six-parameter class of states, and present numerical evidence supporting this conjecture. If our conjecture holds true, then the entanglement potential of all two-mode Gaussian states is exactly equal to $h_0(\cdot )$ of the squeezing of formation. In other words, we find that linear passive optics can always maximise the entanglement of a state up to a threshold value decided by the amount of squeezing present in the state. Our result, thus, connects the resource theories of squeezing and entanglement for two-mode Gaussian states and is primely relevant to quantum information and communication protocols, where squeezed states play a major role.

Our paper is arranged as follows: In Sect. "Background", we discuss some preliminaries of Gaussian quantum information. Then, in Sect. "Saturating the EOF potential" we introduce a special class of potential-saturating Gaussian states, and propose an algorithm to passively transform arbitrary two-mode Gaussian states into potential-saturating states. We present numerical simulations of our algorithm in Sect. "Numerical simulations" to support our conjecture. Finally we conclude in Sect. "Discussion" with a discussion of our results and remarks on future scope.

## Results

### Background

#### Gaussian quantum information

Gaussian quantum states, which are the focus of this work, can be fully described by the second statistical moments of the associated bosonic-field quadrature operators (assuming the first statistical moments, i.e., the mean values, to be zero). The quadrature field operators $\hat{x}_j$ and $\hat{p}_j$ are the real and imaginary parts, respectively, of the bosonic-field annihilation operator for the $j^\text {th}$ mode. Accordingly, any *N*-mode Gaussian state admits a finite-dimensional representation via the covariance matrix $\sigma$ of its quadrature field operators. This covariance matrix is a $2N\times 2N$ real symmetric matrix satisfying the uncertainty relation^29^ $\sigma +i \Omega \ge 0$, where $\Omega$ is the symplectic form given in the Supplemental Material Sect. "Gaussian transformations and their matrix representations". Apart from the regular eigenvalues $\{\lambda _j\}$ of $\sigma$, it is also useful to also define the symplectic eigenvalues $\{\nu _j\}$ of $\sigma$, which are the positive eigenvalues of $i \Omega \sigma$. We denote the symplectic eigenvalues arranged in increasing order by $\nu _j^\uparrow$, so that $\nu _1^\uparrow \le \nu _2^\uparrow \le \nu _N^\uparrow$. Then, the uncertainty relation for $\sigma$ is equivalent to the condition $\nu _1 \ge 1$^2^.

In the symplectic representation, Gaussian transformations, which map Gaussian states to themselves, are given by symplectic matrices $K \in \textrm{Sp} (2 N, \mathbb {R})$, such that $K \Omega K^\top = \Omega$, and *K* acts on $\sigma$ as $\sigma \mapsto K \sigma K^\top$. Here $\textrm{Sp} (2 N, \mathbb {R})$ denotes the group of symplectic $2N\!\times \!2N$ matrices over real numbers. Typical Gaussian transformations include beam splitters $K_\text {bs}(\tau )$ with transmissivity $\tau \in [0,1]$ and phase rotations $K_\text {rot}(\theta )$ with angle $\theta \in [0, 2\pi )$; these are both passive operations, meaning they do not introduce extra energy into the system and thus, leave the trace of the covariance matrix, $\textrm{Tr}\sigma$ invariant.

Active Gaussian transformations, on the other hand, include two local single-mode squeezers, denoted $S_1(r_1, r_2)$, with real-valued squeezing parameters $r_j$ for mode $j\in \{1,2\}$ or two-mode squeezers $S_2(r)$ for *r* the single real squeezing parameter; these transformations introduce extra energy into the system. We summarise these transformations and their matrix representations in the Supplemental Material Sect. "Gaussian transformations and their matrix representations". We also list a few standard decompositions in Gaussian quantum optics in Sect. "Standard decompositions in Gaussian optics" of the Supplemental Material; these will be used later in Sects. "Saturating the EOF potential" and "Numerical simulations".

The covariance matrix $\pi$ of a pure Gaussian state satisfies $\det \pi =1$, whereas for a mixed Gaussian state $\sigma$, we have $\det \sigma > 1$. Such a mixed state $\sigma$ can be decomposed into a pure state $\pi$ and some positive definite matrix, $\phi >0$, representing noise as $\sigma = \pi + \phi$, but this decomposition is not unique. Owing to this non-uniqueness, one way to extend some resource measure $\mathcal {F}$ defined for pure states to mixed states is by optimising over all possible pure state decompositions as follows

1 $$\begin{aligned} \mathcal {F}(\sigma ) :=\min _{\pi }\left\{ \mathcal {F}(\pi ) \, \vert \, \sigma -\pi \ge 0\, , \det \pi =1 \right\} , \end{aligned}$$

where the minimisation is over all pure states $\pi$. Physically, this corresponds to the minimum amount of resource $\mathcal {F}(\pi )$ required to create the state $\sigma$. Below we discuss two resource measures defined in this way—the squeezing of formation $\mathcal {S}(\sigma )$ and the entanglement of formation potential $\mathcal {P}(\sigma )$ of a Gaussian state $\sigma$.

#### Squeezing of formation

The process of squeezing a Gaussian state’s uncertainty below the standard quantum limit^30^, along one quadrature, is an active transformation. Operational measures of squeezing have been proposed^28^ in order to quantify the amount of squeezing in a state. One such measure called the squeezing of formation (SOF), denoted $\mathcal {S}(\sigma )$, is defined as the minimum amount of local squeezing required to construct $\sigma$ starting from vacuum^28^. For an *N*-mode pure Gaussian state $\pi$, this quantity is simply a function of the eigenvalues of $\pi$,

2 $$\begin{aligned} \mathcal {S}(\pi ) :=-\frac{1}{2} \sum _{j=1}^N \ln \left( \lambda _j^{\uparrow } (\pi ) \right) = \frac{1}{2} \sum _{j=N+1}^{2N} \ln \left( \lambda _j^{\uparrow } (\pi ) \right) , \end{aligned}$$

where $\lambda _j^\uparrow (\pi )$ denotes the $j^\text {th}$ lowest eigenvalue of $\pi$. Straightforwardly, the SOF of a two-mode locally-squeezed vacuum with squeezing parameters $r_1$ and $r_2$ is simply $r_1+r_2$. Finally, for mixed states $\sigma$, the SOF definition is then extended via

3 $$\begin{aligned} \mathcal {S}(\sigma ) :=\min _{\pi }\{ \mathcal {S}(\pi ) \, \vert \, \sigma -\pi \ge 0\,, \det \pi =1\}, \end{aligned}$$

where the minimisation is over all pure states $\pi$.

#### Entanglement of formation potential

A two-mode Gaussian $\sigma$ is separable if and only if its partial transpose, denoted $\sigma ^\Gamma$, is also a valid state, i.e.,

4 $$\begin{aligned} \nu _1^\uparrow (\sigma ^\Gamma ) \ge 1 , \end{aligned}$$

a result known as the PPT condition^31^. In this case, $\sigma$ has zero entanglement irrespective of which entanglement measure is employed. However, for mixed entangled states, the various measures of entanglement, including logarithmic negativity^26^, entanglement of formation^27^, distillable entanglement^27^, and relative entropy of entanglement^32^, are all in general inequivalent^33,34^.

We limit our scope to two-mode Gaussian states, which can be treated as a bipartite system, and we choose the entanglement of formation (EOF), denoted $\mathcal {E}(\sigma )$, as our entanglement measure^27^. Conceptually, $\mathcal {E}(\sigma )$ quantifies the minimum amount of entanglement required to produce the state $\sigma$, assisted only by local operations and classical communication (LOCC). For pure states $\pi$, $\mathcal {E}(\pi )$ is defined to be the entropy of entanglement^33,35^, i.e.,

5 $$\begin{aligned} \mathcal {E}(\pi ):=\max \left\{ 0, h\left[ \nu _1^{\uparrow } (\pi ^\Gamma )\right] \right\} , \end{aligned}$$

where $h[\cdot ]$ is an auxiliary function defined in the Supplemental Material Sect. "De-cross-correlated pure states saturating the EOF potential". Then, for mixed states $\sigma$, the definition is extended via Eq. (1) to^36–39^

6 $$\begin{aligned} \mathcal {E}(\sigma ) :=\min _{\pi }\{ \mathcal {E}(\pi ) \, \vert \, \sigma -\pi \ge 0\,, \det \pi =1\}. \end{aligned}$$

Note that Eq. (6) technically defines the Gaussian-EOF^39^, which, in general, upper-bounds the EOF for multi-mode states, but coincides with the EOF for two-mode Gaussian states^40^.

Next, the EOF potential $\mathcal {P}$ is defined as the maximum EOF a state can attain when transformed only by passive linear optics^25^. Specifically, starting from a two-mode Gaussian state $\sigma$, with access to two ancillary vacuum modes, and four-mode passive transformations *K*, the EOF potential is defined as

7 $$\begin{aligned} \mathcal {P}(\sigma ) :=\sup _{K} \left\{ \mathcal {E} ( \sigma ') \, {\bigg \vert } \, \sigma ' = \textrm{tr}_2 \left[ K (\sigma \oplus \mathbbm {1}_2) K^\top \right] \right\} , \end{aligned}$$

so that $\mathcal {E}(\sigma ) \le \mathcal {P}(\sigma )$ always. In Eq. (7), the $\mathbbm {1}_2$ denotes two ancillary vacuum modes and the $\textrm{tr}_2$ denotes tracing out these modes. Intuitively, the EOF potential denotes the maximum EOF we can get from the state $\sigma$ by rearranging it between the four modes, two original modes plus two ancillary modes. Interestingly, $\mathcal {P}(\sigma )$ is upper-bounded by a simple function of $\mathcal {S}(\sigma )$^25^,

8 $$\begin{aligned} \mathcal {P}(\sigma ) \le h_0\left[ \mathcal {S}(\sigma ) \right] , \end{aligned}$$

where $h_0[\cdot ]$ is a monotonic auxiliary function defined in the Supplemental Material Sect. "De-cross-correlated pure states saturating the EOF potential". However, the saturability of the bound in Eq. (8) for arbitrary $\sigma$ remains an open problem. In this work, we provide an algorithm that aims to saturate this bound for arbitrary two-mode Gaussian states and then establish this saturability via extensive numerical testing.

### Saturating the EOF potential

In this section, we first introduce a special class of potential-saturating two-mode Gaussian states, $\sigma _{\textrm{sp}}$ ($\textrm{sp}$ for special), which have $\mathcal {E}(\sigma _{\textrm{sp}})=\mathcal {P}(\sigma _{\textrm{sp}})=h_0\left[ \mathcal {S}(\sigma _{\textrm{sp}})\right]$, and thus saturate the bound in Eq. (8). We state this claim as a proposition and then prove it in Sect. " A special class of states". Then, in Sect. " Extension to all two-mode Gaussians", we conjecture that any arbitrary two-mode Gaussian state can be passively transformed into this special class. In Sect. " Algorithm: Passive operations to maximise EOF" we provide an explicit algorithm to perform this transformation. If our conjecture holds true, then $\mathcal {P}(\sigma ) = h_0\left[ \mathcal {S}(\sigma ) \right]$ for all two-mode Gaussian states.

#### A special class of states

Consider the two-mode Gaussian state

9 $$\begin{aligned} \sigma _\text {sp} =K_\text {bs} \big ( \pi _d(r_1,r_2)+\lambda _1\phi _1+\lambda _2\phi _2\big ) K_\text {bs}^\top \,, \end{aligned}$$

where $\pi _\text {d}(r_1,r_2)$ represents a locally-squeezed two-mode pure state in diagonal form with squeezing parameters $r_1$ and $r_2$ (matrix representation in Supplemental Material Sect. "Gaussian transformations and their matrix representations"). Here $K_\text {bs}$ denotes a balanced beam splitter operation with $\tau =1/2$, $\lambda _2 \ge \lambda _1 \ge 0$ are two non-negative constants, and $\phi _1=|{\phi _1}\rangle \langle {\phi _1}|$ and $\phi _2=|{\phi _2}\rangle \langle {\phi _2}|$ are two orthogonal, positive semidefinite, rank-one matrices with

10 $$\begin{aligned} \left| \phi _1\right\rangle =\begin{pmatrix} \alpha \cos \theta \\ \sin \theta \\ \cos \theta \\ \alpha \sin \theta \end{pmatrix}\;\text {and}\; \left| \phi _2\right\rangle =\begin{pmatrix} \alpha \sin \theta \\ cos\theta \\ \sin \theta \\ - \alpha \cos \theta \end{pmatrix}\,. \end{aligned}$$

In Eq. (10), $\alpha$ is a real parameter satisfying $\vert \alpha \vert \le e^{-r_1-r_2}$ and $\theta \in [0, 2\pi )$ is an angle. The term $\lambda _1 \phi _1 + \lambda _2 \phi _2$ can be thought of as correlated noise, parameterised by $\lambda _1$, $\lambda _2$, $\alpha$ and $\theta$, added to the pure two-mode squeezed state $\pi _d$. The terms $\lambda _1$ and $\lambda _2$ denote the strength of the noise terms $\phi _1$ and $\phi _2$, respectively. The parameter $\alpha$ determines the ratio between the added noise in the first and the second modes in the same quadrature, whereas the angle $\theta$ determines the ratio between the added noise in the $\hat{x}$ and $\hat{p}$ quadratures in the same mode. When $\lambda _1=\lambda _2$, the form of the added noise $\lambda _1 \phi _1 +\lambda \phi _2$ is special in the sense that the state $\sigma _{\textrm{sp}}$ becomes passively de-cross-correlatable, i.e., can be passively transformed into a de-cross-correlated state (recall that de-cross-correlated states have no correlations between the $\hat{x}$ and $\hat{p}$ quadratures, i.e., $\langle \hat{x}_i \hat{p}_j + \hat{p}_i \hat{x}_j \rangle =0$ for $i, j \in \{1, 2\}$, see Supplemental Material Sect. "Gaussian transformations and their matrix representations" for details). Overall, the state $\sigma _{\textrm{sp}}$ has 6 free parameters $\{r_1, r_2, \lambda _1, \lambda _2, \alpha , \theta \}$ and thus may be thought of as an element from a six-parameter family of states.

As we shall show in the following proposition, the state $\sigma _{\textrm{sp}}$ is special in the sense that: $\sigma _{\textrm{sp}}$ has the same SOF as $\pi _d$, the EOF of $\sigma _{\textrm{sp}}$ saturates its EOF potential and $\sigma _{\textrm{sp}}$ has the same EOF potential as $\pi _d$:

11 $$\begin{aligned} \mathcal {S}(\sigma _\text {sp}) =\mathcal {S}(\pi _d) \; \; \text {and} \; \; \mathcal {E}(\sigma _\text {sp}) = \mathcal {P}(\sigma _\text {sp}) = \mathcal {P}(\pi _d). \end{aligned}$$

Moreover, the EOF and SOF properties of a pure state $\pi _\text {d}$ are simply

12 $$\begin{aligned} \mathcal {S}(\pi _\text {d}) = r_1+r_2\quad \text {and}\quad \mathcal {P}(\pi _\text {d}) = h_0 \left[ \mathcal {S}(\pi _d) \right] . \end{aligned}$$

In other words, the upper bound for EOF in Eq. (8) is saturated for all such $\sigma _{\textrm{sp}}$. We now formally state and prove this claim.

**Figure 1:**
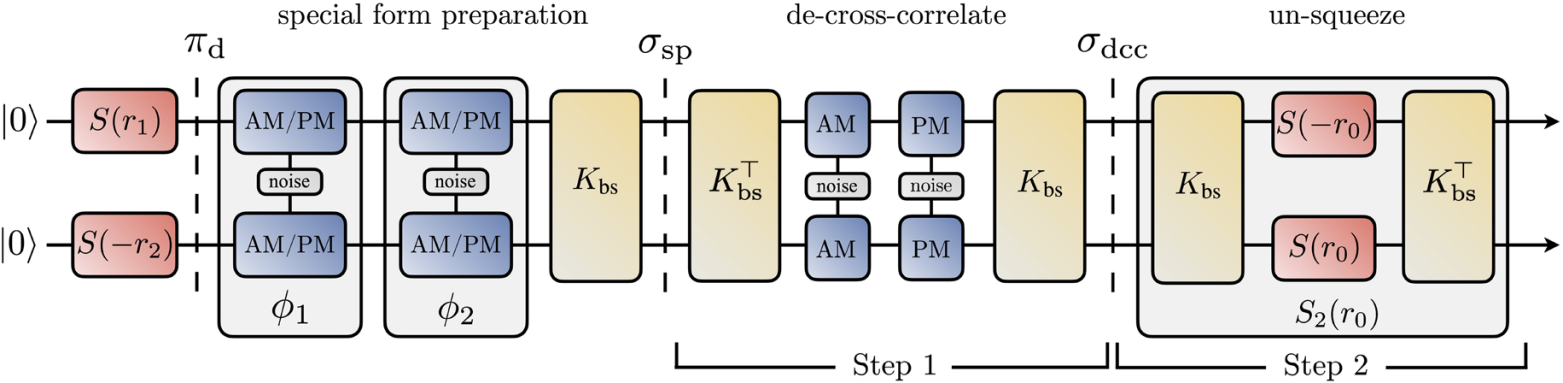
Schematic of the procedure to compute the EOF potential $\mathcal {P}$ for a state $\sigma _\text {sp}$ in the special form given in Eq. (9). Steps 1 and 2 from the proof of our proposition are also indicated. After adding a particular correlated noise to $\sigma _{\textrm{sp}}$ (step 1), the de-cross-correlated state $\sigma _\mathrm{dcc}$ is then two-mode-squeezed to produce a separable state (step 2). The minimum value $r_0$ of the two-mode squeezing parameter, such that the output state is separable, yields the lower bound $h_0[2 r_0]$ to $\mathcal {P}(\sigma _{\textrm{sp}})$, as in Eq. (17).

##### Proposition

For any state $\sigma _{\textrm{sp}}$ of the form in Eq. (9), the EOF upper bound in Eq. (8) is saturated, i.e.,

13 $$\begin{aligned} \mathcal {E}(\sigma _{\textrm{sp}}) = \mathcal {P}(\sigma _{\textrm{sp}}) =h_0 \left[ \mathcal {S}(\sigma _{\textrm{sp}})\right] \; . \end{aligned}$$

##### Proof

The outline of our proof is as follows. By adding classical correlations in the form of noise to $\sigma _{\textrm{sp}}$, we get a state $\sigma _\mathrm{dcc}$ that is de-cross-correlated. We then lower-bound $\mathcal {E}(\sigma _\mathrm{dcc})$, which serves as a lower bound for $\mathcal {E}(\sigma _{\textrm{sp}})$ and thus $\mathcal {P}(\sigma _{\textrm{sp}})$. Finally, we upper-bound $\mathcal {S}(\sigma _{\textrm{sp}})$ and show that this upper bound coincides with the lower bound for $\mathcal {P}$, which along with Eq. (8) implies that $\mathcal {P}(\sigma _{\textrm{sp}}) = h_0\left[ \mathcal {S}(\sigma _{\textrm{sp}})\right]$. The proof presented below is broken up into three steps, and is illustrated in Fig. 1 as a circuit diagram.

Step 1: We first add some noise along $K_\mathrm{bs} \phi _1 K_\mathrm{bs}^\top$ to $\sigma _{\textrm{sp}}$ to get a de-cross-correlated state $\sigma _\mathrm{dcc}$,

$$\begin{aligned} \sigma _\mathrm{dcc} =\sigma _\text {sp}+(\lambda _2-\lambda _1)K_\text {bs} \phi _1 K_\text {bs}^\top = K_\text {bs}(\pi _\text {d} + \lambda _2(\phi _1+\phi _2))K_\text {bs}^\top = K_\text {bs} \left( \pi _d + \lambda _2 \begin{pmatrix}\alpha ^2&{}0&{}\alpha &{}0\\ 0&{}1&{}0&{}\alpha \\ \alpha &{}0&{}1&{}0\\ 0&{}\alpha &{}0&{}\alpha ^2\end{pmatrix} \right) K_\text {bs}^\top \;. \end{aligned}$$

As adding noise cannot increase entanglement, we have

14 $$\begin{aligned} \mathcal {E}(\sigma _\mathrm{dcc}) \le \mathcal {E}(\sigma _\text {sp}). \end{aligned}$$

Step 2: Next, we consider the least amount of two-mode squeezing, $r_0$, required to un-squeeze $\sigma _\mathrm{dcc}$ into a separable state, i.e.,

15 $$\begin{aligned} r_0 :=\min _{r}\left\{ r \Bigm | S_2(r) \sigma _\mathrm{dcc} S_2^\top (r) \;\text {separable}\right\} \;. \end{aligned}$$

Then $h_0\left[ 2 r_0\right]$ is a lower bound to $\mathcal {E}(\sigma _\mathrm{dcc})$. By checking the necessary and sufficient conditions for separability (see Sect. “Background”), we find that the state $S_2(r)\sigma _\mathrm{dcc} S_2^\top (r)$ is separable when

16 $$\begin{aligned} \frac{r_1 + r_2}{2} \le r \le \frac{1}{4}\sum _{j=1}^2 \log \left[ \frac{\lambda _2+e^{2r_j}}{1+\lambda _2 \alpha ^2 e^{2r_j}} \right] , \end{aligned}$$

so that $r_0 = (r_1+r_2)/2$. Moreover, for the interval in Eq. (16) to be valid, we must have $\vert \alpha \vert \le e^{-r_1-r_2}$. The lower bound $h_0[2 r_0] = h_0[r_1+r_2] \le \mathcal {E}(\sigma _\mathrm{dcc})$ from Eq. (16), when combined with Eq. (14), results in

17 $$\begin{aligned} h_0[r_1+r_2] \le \mathcal {E}(\sigma _\mathrm{dcc}) \le \mathcal {E}(\sigma _\text {sp}) \le \mathcal {P}(\sigma _\text {sp}) . \end{aligned}$$

Step 3: Finally, we observe that $\sigma _{\textrm{sp}}$ can clearly be produced with $r_1+r_2$ amount of squeezing, so that $\mathcal {S}(\sigma _\text {sp})\le r_1+r_2$. The monotonicity of $h_0(\cdot )$ and Eq. (8) then allows us to upper-bound $\mathcal {E}(\sigma _{\textrm{sp}})$ as

18 $$\begin{aligned} \mathcal {E}(\sigma _\text {sp})\le \mathcal {P}(\sigma _{\textrm{sp}}) \le h_0 \left[ \mathcal {S}(\sigma _\text {sp})\right] \le h_0[r_1+r_2]. \end{aligned}$$

Combining Eqs. (17) and (18), we get

19 $$\begin{aligned} \mathcal {E}(\sigma _\text {sp})=\mathcal {P}(\sigma _\text {sp})=h_0[r_1+r_2]\;\text {and}\;\mathcal {S}(\sigma _\text {sp})=r_1+r_2 \,, \end{aligned}$$

thus proving the proposition. $\square$

The proposition above says that for states in the special form of Eq. (9), the upper bound $h_0\left[ \mathcal {S}(\cdot )\right]$ (introduced in Ref.^25^) on the EOF potential $\mathcal {P}(\cdot )$ is actually the true value of $\mathcal {P}(\cdot )$. In other words, all states in this six-parameter family saturate the inequality in Eq. (8). Notably, previously, only two three-parameter families of two-mode Gaussian states were known to possess this property: symmetric states and balanced correlated states^25^.

#### Extension to all two-mode Gaussians

Let us now denote by *G* the set of all states in the special form of Eq. (9). Suppose a state $\sigma '$ is not in this set *G*, but on applying some passive transformation $K'$ transforms into the special form, i.e.,

20 $$\begin{aligned} \sigma '\not \in G \quad \textrm{but} \quad K' \sigma ' K'^\top \in G. \end{aligned}$$

As passive transformations by definition do not change the EOF potential of a state^25^, we must have

21 $$\begin{aligned} \mathcal {P}(\sigma ')= \mathcal {P}(K'\sigma 'K'^\top ) = h_0 \left[ \mathcal {S}(K'\sigma 'K'^\top ) \right] . \end{aligned}$$

Moreover, passive transformations also leave the SOF invariant^28^, so $\mathcal {S}(K'\sigma 'K'^\top ) = \mathcal {S}(\sigma ')$. Thus, we have

22 $$\begin{aligned} \mathcal {P}(\sigma ') = h_0 \left[ \mathcal {S}(\sigma ')\right] , \end{aligned}$$

indicating that $\sigma '$ too saturates the inequality in Eq. (8) despite not being in the set *G*. By a similar line of reasoning, it follows that for any state $\sigma '\not \in G$, if we can add some noise $\phi '$ such that its SOF remains unchanged, i.e., $\mathcal {S}(\sigma ') = \mathcal {S}(\sigma '+\phi ')$, and the resulting state is in the special form, i.e., $\sigma '+\phi ' \in G$, then $\sigma '$ must also satisfy Eq. (22).

It is then evident that any state that can be transformed into *G* by either passive transformations, or the addition of noise that keeps the SOF constant, or both, must also saturate the upper bound in Eq. (8). We conjecture that all two-mode Gaussian states can be transformed into *G* in this way.

##### Conjecture

Any two-mode Gaussian state $\sigma _\mathrm{in}$ can be transformed into some element $\sigma _\mathrm{out}$ in *G*, without increasing its SOF, via only passive transformations, the addition of noise and access to ancillary vacuum modes.

From the discussion in Sect. "A special class of states" we know that our conjecture, if true, would immediately imply that $\mathcal {P}(\sigma _\mathrm{in})=h_0\left[ \mathcal {S}(\sigma _\mathrm{in})\right]$ for all two-mode Gaussian states $\sigma _\mathrm{in}$. In this work, we do not formally prove our conjecture—instead, we provide evidence for the conjecture in the following way. First we present the transformation $\sigma _\mathrm{in} \mapsto \sigma _\mathrm{out}$ mentioned in the conjecture as an algorithm (Algorithm 1 in Sect. "Algorithm: Passive operations to maximise EOF"). Algorithm 1 takes $\sigma _\mathrm{in}$ as input, and after performing passive operations, adding noise, and adding and then discarding an ancillary vacuum mode, the algorithm outputs the transformed state $\sigma _\mathrm{out} \in G$. Next, we numerically ran our algorithm on $10^6$ random inputs $\sigma _\mathrm{in}$, and calculate the EOF of the output $\mathcal {E}(\sigma _\text {out})$ and compare that to the SOF of the input $\mathcal {S}(\sigma _\text {in})$. We verify that  $\mathcal {E}(\sigma _\mathrm{out})=h_0[ \mathcal {S}(\sigma _\mathrm{in})]$ holds true for every input state to within numerical tolerances.

#### Algorithm: passive operations to maximise EOF

We now propose an algorithm that, starting from any arbitrary two-mode Gaussian $\sigma _\mathrm{in}$, outputs a potential-saturating two-mode Gaussian $\sigma _\mathrm{out}$ such that $\mathcal {E}(\sigma _\mathrm{out})=\mathcal {P}(\sigma _\mathrm{out})=h_0 \left[ \mathcal {S}(\sigma _\mathrm{out}) \right]$ while keeping the SOF constant, i.e., $\mathcal {S}(\sigma _\text {out})=\mathcal {S}(\sigma _\text {in})$. In doing so, the algorithm only performs passive operations and adds noise to the input state so that $\mathcal {P}(\sigma _\mathrm{out}) \le \mathcal {P}(\sigma _\mathrm{in})$. As a result, our algorithm establishes the fact that $\mathcal {P}(\sigma _\mathrm{in})= h_0 \left[ \mathcal {S}(\sigma _\mathrm{in})\right]$ for any arbitrary two-mode Gaussian $\sigma _\mathrm{in}$. The fundamental idea behind the algorithm is to decouple the squeezing between the two modes of $\sigma _\mathrm{in}$, and then mix the two modes on a balanced beam splitter. The resulting de-cross-correlated state, with two identical modes, is known to be potential-saturating and also saturates the EOF bound in Eq. (8) (see Supplemental Material Sect. "De-cross-correlated pure states saturating the EOF potential").

The first step in the algorithm is to find an optimal pure state $\pi _\mathrm{opt}$ that has the same SOF as $\sigma _\mathrm{in}$ from Eq. (3), i.e., $\mathcal {S}(\sigma _\mathrm{in}) = \mathcal {S}(\pi _\mathrm{opt})$; in Algorithm 1, we denote this procedure as OptSOFState$(\sigma _\mathrm{in})$^28^. Next, BMDecomp$(\pi _\mathrm{opt})$ leverages the Bloch-Messiah decomposition to find a passive transformation $K_\mathrm{BM}$ that that diagonalises $\pi _\mathrm{opt}$ to $\pi _\mathrm{diag}$ (see Supplemental Material Sect. "Standard decompositions in Gaussian optics" for details). Applying $K_\mathrm{BM}$ to the mixed state $\sigma _{\textrm{in}}=\pi _\mathrm{opt}+\phi$ yields the mixed state $\sigma _{\textrm{diag}}=\pi _\mathrm{diag} + \phi _\mathrm{diag}$ (note that $\sigma _{\textrm{diag}}$ and $\phi _{\textrm{diag}}$ are not diagonal). In the second step, we calculate the eigenvalues $\{\lambda _j\}$ (arranged in decreasing order) and eigenvectors $\{\left| \phi _j\right\rangle \}$ of the matrix $\phi _\mathrm{diag}$ via the procedure Spectrum$(\phi _\mathrm{diag})$. Then we compute the extra noise term $\phi _\mathrm{extra} = (\lambda _1 - \lambda _2) |{\phi _2}\rangle \langle {\phi _2}|$, which, when added to $\sigma _\mathrm{diag}$, gives us the state $\sigma '=\sigma _\mathrm{diag}+\phi _\mathrm{extra}$.

Surprisingly, we find that the state $\sigma '$ at this point in the algorithm can always be passively de-cross-correlated. This is not true, in general, for mixed Gaussian states. Nevertheless, for all $\sigma _\mathrm{in}$, $K_\mathrm{BM} \sigma _\mathrm{in} K_\mathrm{BM}^\top + \phi _\mathrm{extra}$ becomes a passively de-cross-correlatable state—this is crucial because de-cross-correlated states are optimal for the EOF potential (see Supplemental Material Sect. "De-cross-correlated pure states saturating the EOF potential"). This passive transformation, which is simply a phase rotation on one mode, is calculated in the procedure DeCrossCorrelate$(\cdot )$ by numerically finding the angle $\theta ^*\in [0, 2 \pi )$ and mode $i^*\in \{1, 2\}$ to be rotated to make $\sigma '$ de-cross-correlated. The last step in the algorithm comprises mixing one of the modes of the de-cross-correlated state $\sigma _\mathrm{rot}$ with a third ancillary vacuum mode on a beam splitter; this is done to remove noise from $\sigma _\mathrm{rot}$. The transmissivity $\tau ^*\in [0, 1]$ for this beam splitter operation $K_\mathrm{bs}^{3, j^*}$ and the mode $j^*\in \{1,2\}$ to be mixed with vacuum are calculated numerically by maximising the EOF of the resulting state. Details of the numerical procedure for calculating EOF are presented in Sect. "Numerical simulations". This final state is output as $\sigma _\mathrm{out}$ by Algorithm 1, which we present below in full.
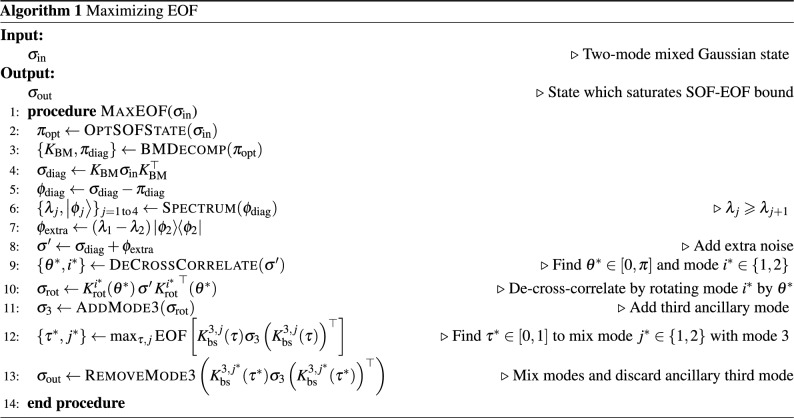


We note that for states $\sigma _\mathrm{in}$ with both modes squeezed, steps 5 through to 13 may be skipped in Algorithm 1, and instead a final balanced beam splitter $K_\mathrm{bs}$ suffices to bring $\sigma _\mathrm{in}$ into *G*. More precisely,

23 $$\begin{aligned} \sigma _\mathrm{out} = K_\mathrm{bs} K_\mathrm{BM} \sigma _\mathrm{in} K_\mathrm{BM}^\top K_\mathrm{bs} ^\top \in G. \end{aligned}$$

Thus $K_\mathrm{bs} K_\mathrm{BM}$ is the passive transformation that maximizes the EOF of $\sigma _\mathrm{in}$, or, alternatively, transforms $\sigma _\mathrm{in}$ into the set *G*.

### Numerical simulations

**Figure 2:**
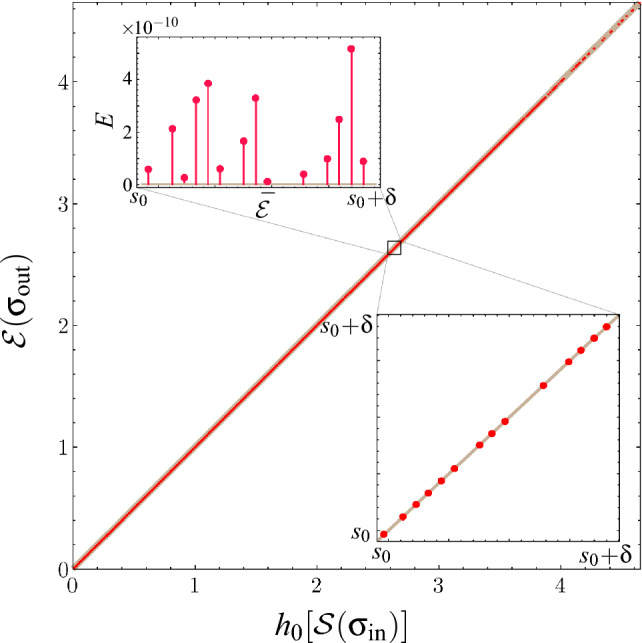
Numerical results from running Algorithm 1 on a million random two-mode Gaussian states. The output state’s $\mathcal {E}$ and the input state’s $h_0(\mathcal {S})$ values coincide (red dots) and, thus, lie on the $Y=X$ line (thick, gray) to within numerical tolerance. The bottom inset magnifies the section $[s_0, s_0+\delta ]$ (where $s_0=2.6430777$ and $\delta =4.1\!\times \!10^{-6}$) of the main plot. The top inset rotates this same section, by plotting the error $E=\mathcal {E}-h_0[\mathcal {S}]$ against $\bar{\mathcal {E}}=(\mathcal {E}+h_0[\mathcal {S}])/2$. Over a million runs, the average absolute error $\vert E \vert _{\textrm{avg}}$ is $1.93\!\times \!10^{-9}$.

In order to support our conjecture, we numerically apply Algorithm 1 to $10^6$ randomly generated two-mode Gaussian states. This random generation leverages Williamson’s decomposition (see Supplemental Material Sect. "Standard decompositions in Gaussian optics") by applying random active and passive operations on randomly generated two-mode thermal states. For each randomly generated instance, its SOF and the corresponding optimum pure state is computed numerically, based on an algorithm provided in Ref.^28^ with a numerical accuracy of $10^{-8}$. Then, this state is transformed according to Algorithm 1, and the EOF of the output state is calculated. For arbitrary two-mode Gaussian states, there are several equivalent approaches (but no simple analytical expression) to calculate the Gaussian EOF^33,36,38,39,41^. We used the approach from Ref.^33^ to compute Gaussian EOFs in this work.

By testing on $10^6$ such randomly generated two-mode Gaussian states, we see that the difference between the EOF $\mathcal {E}(\sigma _\mathrm{out})$ and the upper bound $h_0\left[ \mathcal {S}\left( \sigma _\mathrm{in}\right) \right]$ is always lower than numerical tolerance. The average absolute error $\left| \mathcal {E}-h_0[\mathcal {S}] \right|$ over a million runs is $1.93\!\times \!10^{-9}$.

We also explicitly verify that Algorithm 1 does not change the SOF of the input state, i.e., $\mathcal {S}(\sigma _\mathrm{in}) = \mathcal {S}(\sigma _\mathrm{out})$. The results from this test are shown in Fig. 2, where the straight line plot between $\mathcal {E}$ and $h_0[\mathcal {S}]$ provides strong evidence supporting our conjecture.

Based on our proposition, and the numerical results supporting our conjecture shown in Fig. 2, it follows that the EOF potential of all two-mode Gaussian states is a monotonic function of the state’s SOF. Qualitatively, this means the maximum EOF, when restricted to linear passive optics, is completely determined by the minimum amount of local squeezing required for state preparation. Conversely, to increase EOF beyond this value, further squeezing operations are necessarily required.

## Discussion

In this work, we have studied the relation between the squeezing of formation and the maximum entanglement of formation under passive operations for two-mode Gaussian states. We have characterised a special six-parameter family of two-mode states, which are potential-saturating and also saturate the SOF-EOF bound. Moreover, we have conjectured that any arbitrary two-mode Gaussian state can be passively transformed into the aforementioned family. In support of our conjecture, we have proposed an algorithm to passively transform arbitrary two-mode Gaussian states into this special class. Finally, we report numerical results from simulating this algorithm on a million random instances, which supports our conjecture.

In conclusion, we claim that the entanglement potential for all two-mode Gaussian states is completely determined by the minimum amount of squeezing required to construct the state. By connecting an operational measure for squeezing to one for entanglement, our work establishes a satisfying link between the resource theories of squeezing and entanglement. Furthermore, being restricted solely to passive linear optics, the steps in our proposed algorithm are practically feasible in experimental setups. As an example application, our results could be used to quantify and compare the entangling capabilities of different experimental setups.

Our work draws a natural conclusion to the line of research investigating the relationship between entanglement potential and squeezing for two-mode Gaussian states. As both these quantities can be extended to multi-mode states, the validity of the SOF-EOF bound and its saturability remain open problems in the greater-than-two-mode case. Notably, in this case, the Gaussian EOF and the EOF do not coincide so the entanglement potential must be redefined carefully^39,40^.

## Supplementary Information

Supplementary Information.

## Data Availability

The data that support the findings of this study are available from the corresponding author upon reasonable request.

## Code availability

The codes that support the findings of this study are available from the corresponding author upon reasonable request.
