# Supplementary material for: On the equivalence between squeezing and entanglement potential for two-mode Gaussian states
Source: Sci Rep. 2023 Jul 20;13:11722. doi: 10.1038/s41598-023-38572-1 (PMC10359327; doi:10.1038/s41598-023-38572-1)
Supplement: Supplementary file 1 — Supplementary Information. [file 41598_2023_38572_MOESM1_ESM.pdf]

Supplementary Material: On the equivalence between squeezing and entanglement potential for two-mode Gaussian states

Bohan Li[1,+], Aritra Das[1,+], Spyros Tserkis[2], Prineha Narang[2], Ping Koy Lam[1,4], and Syed M. Assad[1,3,*]

[1]Centre for Quantum Computation and Communication Technology, Department of Quantum Science and Technology, Australian National University, Canberra, ACT 2601, Australia
[2]Physical Sciences, College of Letters and Science, University of California, Los Angeles (UCLA), CA, USA
[3]School of Physical and Mathematical Sciences, Nanyang Technological University, Singapore 639673, Republic of Singapore
[4]Institute of Materials Research and Engineering, Agency for Science, Technology and Research (A*STAR), Singapore 138634, Republic of Singapore
[*]cqtsma@gmail.com
[+]these authors contributed equally to this work

1 Gaussian transformations and their matrix representations

In this section, we briefly review some common Gaussian transformations. Gaussian transformations are represented by symplectic matrices S, which satisfy $S\Omega S^\top = \Omega$, where

$$\Omega = \bigoplus_{j=1}^{N} \begin{pmatrix} 0 & 1 \\ -1 & 0 \end{pmatrix} \tag{1}$$

is the symplectic form. Two-mode beam splitters are represented by

$$K_{\mathrm{bs}}(\tau) = \begin{pmatrix} \sqrt{\tau}\,\mathbb{1}_2 & \sqrt{1-\tau}\,\mathbb{1}_2 \\ -\sqrt{1-\tau}\,\mathbb{1}_2 & \sqrt{\tau}\,\mathbb{1}_2 \end{pmatrix}, \tag{2}$$

where $\mathbb{1}_N$ represents the identity operator on N dimensions. For balanced beam splitters, where $\tau = 1/2$, we drop the explicit dependence on τ and simply write K_{bs}. Single-mode phase rotations are represented via the rotation matrices,

$$K_{\mathrm{rot}}(\theta) = \begin{pmatrix} \cos\theta & \sin\theta \\ -\sin\theta & \cos\theta \end{pmatrix}. \tag{3}$$

Beam splitters and phase rotations constitute the fundamental passive linear transformations, as can be seen, e.g., from the rectangular decomposition for passive Gaussian operations (see Sec. 2).

A single-mode squeezer is represented by

$$S(r) = \begin{pmatrix} e^{-r} & 0 \\ 0 & e^{r} \end{pmatrix}, \tag{4}$$

whereas a two-mode squeezer can either be local, as in $S_1(r_1, r_2) := S(r_1) \oplus S(-r_2)$, wherein two single-mode squeezers act independently on two modes indexed by $j \in \{1, 2\}$, or non-local, as in

$$S_2(r) := K_{\mathrm{bs}}\, S_1(-r, r)\, K_{\mathrm{bs}}^\top = K_{\mathrm{bs}} \begin{pmatrix} e^{r} & 0 & 0 & 0 \\ 0 & e^{-r} & 0 & 0 \\ 0 & 0 & e^{-r} & 0 \\ 0 & 0 & 0 & e^{r} \end{pmatrix} K_{\mathrm{bs}}^\top. \tag{5}$$

The locally-squeezed two-mode diagonal Gaussian state π_{d} is

$$\pi_{\mathrm{d}}(r_1, r_2) = S_1(r_1, r_2)\mathbb{1}_4 S_1(r_1, r_2)^\top = \begin{pmatrix} e^{-2r_1} & 0 & 0 & 0 \\ 0 & e^{2r_1} & 0 & 0 \\ 0 & 0 & e^{2r_2} & 0 \\ 0 & 0 & 0 & e^{-2r_2} \end{pmatrix}. \tag{6}$$

For convenience, we sometimes drop the r_1, r_2 dependence and write simply π_{d}. When restricted to two-mode Gaussian states, the Bloch-Messiah decomposition[1,2] states that phase rotations, beam splitters and single- and two-mode squeezers are sufficient to implement arbitrary Gaussian transformations.

Finally, for a special subset of two-mode Gaussian states called de-cross-correlated states[3,4] that satisfy $\langle \hat{x}_i \hat{p}_j + \hat{p}_i \hat{x}_j \rangle = 0$ for $i, j \in \{1,2\}$, the covariance matrix σ takes the form

$$\sigma = \begin{pmatrix} \sigma_{11} & 0 & \sigma_{13} & 0 \\ 0 & \sigma_{22} & 0 & \sigma_{24} \\ \sigma_{13} & 0 & \sigma_{33} & 0 \\ 0 & \sigma_{24} & 0 & \sigma_{44} \end{pmatrix} = \begin{pmatrix} \sigma_{11} & \sigma_{13} \\ \sigma_{13} & \sigma_{33} \end{pmatrix} \oplus \begin{pmatrix} \sigma_{22} & \sigma_{24} \\ \sigma_{24} & \sigma_{44} \end{pmatrix} =: C_q \oplus C_p, \tag{7}$$

where in the second equality we have re-indexed the usual $[\hat{x}_1, \hat{p}_1, \hat{x}_2, \hat{p}_2]$ operators as $[\hat{x}_1, \hat{x}_2, \hat{p}_1, \hat{p}_2]$. Note that the form in Eq. (7) is also referred to as a standard form[5] and a large class of two-mode Gaussian states can be passively transformed into this form[3]. Moreover, for pure states in this standard form, $C_p = C_q^{-1}$ (the superscript $^{-1}$ denoting matrix inverse), so that $\pi = C_q \oplus C_q^{-1}$ and consequently, $\mathcal{E}(\pi)$ becomes a monotonically increasing function of the sub-correlation matrix C_q[6,7].

2 Standard decompositions in Gaussian optics

In this section, we briefly overview some mathematical decompositions of Gaussian states and operations. The first, known as Williamson's decomposition, provides a way to write arbitrary mixed state σ as a Gaussian (symplectic) transformation S performed on a thermal state represented by diagonal matrix D, i.e.,

$$\sigma = SDS^{\top}, \tag{8}$$

where

$$D := \mathrm{Diag}\left(\nu_1, \nu_1, \ldots, \nu_N, \nu_N\right), \tag{9}$$

and

$$\{\nu_j\} := \left|\mathrm{Eigs}\left(i\Omega\sigma\right)\right| \tag{10}$$

are the symplectic eigenvalues. Here, $\mathrm{Diag}(x)$ denotes a diagonal matrix with diagonal entries given by x and $\mathrm{Eigs}(A)$ denotes the eigenvalues of a matrix A.

The second decomposition, known as the Bloch-Messiah decomposition or Euler decomposition, presents a way to decompose arbitrary Gaussian (symplectic) transformation S into a sequence of a passive (orthogonal) transformation K_1, followed by single-mode squeezing operations on each mode (represented by diagonal matrix $Z = \oplus_{j=1}^{N} S(r_j)$), and a second passive transformation K_2, i.e.,

$$S = K_1 Z K_2 \,. \tag{11}$$

One useful application of the Bloch-Messiah decomposition is in passively diagonalising a pure Gaussian π. Being a pure state, π can be written as $\pi = SS^{\top}$ for S some symplectic matrix. The Bloch-Messiah decomposition then yields $S = K_1 Z K_2$, which results in

$$\pi = K_1 Z Z^{\top} K_1^{\top} = K_1 Z^2 K_1^{\top}. \tag{12}$$

Evidently, $K_1^{\top} \pi K_1 = Z^2$, which is a diagonal matrix, so the passive operation that diagonalises π is $K_1^{\top}$.

Third, the rectangular decomposition allows us to decompose arbitrary passive (i.e., real, orthogonal, and symplectic) transformations into as a sequence of $N(N-1)/2$ beam splitters and single-mode phase rotations. Mathematically, this implies that beam-splitters and single-mode phase rotations generate the set of passive Gaussian transformations.

Lastly, the polar decomposition equates any symplectic matrix S with the product of an orthogonal, symplectic matrix K (representing a passive operation) and a symmetric, positive semidefinite, symplectic matrix P, i.e.,

$$S = KP. \tag{13}$$

This decomposition is unique and plays a crucial role in Gaussian quantum optics[8].

3 De-cross-correlated pure states saturating the EOF Potential

In this section, we establish necessary and sufficient conditions for a de-cross-correlated pure state π_{dcc} to be potential-saturating, i.e., $\mathcal{E}(\pi_{\text{dcc}}) = \mathcal{P}(\pi_{\text{dcc}})$. In the process, we also compute the SOF of this state and find that the potential-saturating state also saturates the upper bound

$$\mathcal{P}(\sigma) \leq h_0\left[\mathcal{S}(\sigma)\right], \tag{14}$$

meaning that

$$\mathcal{E}(\pi_{\text{dcc}}) = \mathcal{P}(\pi_{\text{dcc}}) = h_0\left[\mathcal{S}(\pi_{\text{dcc}})\right] \tag{15}$$

when π_{dcc} satisfies the necessary and sufficient conditions.

As stated previously, the covariance matrix of a de-cross-correlated pure state π_{dcc} takes the form

$$\pi_{\text{dcc}} = C_q \oplus {C_q}^{-1} = \begin{pmatrix} q_1 & q_3 \\ q_3 & q_2 \end{pmatrix} \oplus \begin{pmatrix} q_1 & q_3 \\ q_3 & q_2 \end{pmatrix}^{-1}, \tag{16}$$

and the uncertainty relation for π_{dcc} simplifies to $q_1 q_2 - q_3^2 \geq 0$ and $q_1, q_2 > 0$. As shown in the discussion on the Bloch-Messiah decomposition in Sec. 2, any pure state can be passively transformed into a diagonal state, and hence a de-cross-correlated state.

For the de-cross-correlated pure state in Eq. (16), the two largest eigenvalues $\lambda_3^{\uparrow}$ and $\lambda_4^{\uparrow}$ can be computed as

$$\lambda_3^{\uparrow} = \frac{q_1 + q_2 + \sqrt{(q_1 - q_2)^2 + 4q_3^2}}{2} \qquad \text{and} \qquad \lambda_4^{\uparrow} = \frac{q_1 + q_2 + \sqrt{(q_1 - q_2)^2 + 4q_3^2}}{2(q_1 q_2 - q_3^2)}, \tag{17}$$

so that the SOF of the state is

$$\mathcal{S}(\pi_{\text{dcc}}) = \frac{1}{2}\ln\left(\lambda_3^{\uparrow}\lambda_4^{\uparrow}\right) = \ln\left(\frac{q_+ + \sqrt{{q_+}^2 - 4\Delta}}{2\sqrt{\Delta}}\right) = \ln\left(\sqrt{\frac{{q_+}^2}{4q_1q_2}\frac{q_1q_2}{\Delta}} + \sqrt{\frac{{q_+}^2}{4q_1q_2}\frac{q_1q_2}{\Delta} - 1}\right). \tag{18}$$

Here we have defined $q_+ := q_1 + q_2$ and $\Delta := \det(C_q)$ in the second equality and rearranged terms in the third. Note that $\mathcal{S}(\pi_{\text{dcc}})$ is a monotonically increasing function of q_+. Using the AM-GM inequality $q_+ \geq 2\sqrt{q_1 q_2}$ and the monotonicity of logarithms, we get the inequality

$$\mathcal{S}(\pi_{\text{dcc}}) \geq \ln\left(\sqrt{\frac{q_1q_2}{\Delta}} + \sqrt{\frac{q_1q_2}{\Delta} - 1}\right), \tag{19}$$

where the equality can hold if and only if $q_1 = q_2$. Next we define $m(C_q) := q_1q_2/\Delta$, so that Eq. (19) becomes

$$\mathcal{S}(\pi_{\text{dcc}}) \geq \ln\left(\sqrt{m(C_q)} + \sqrt{m(C_q) - 1}\right) =: \mathcal{S}_0(\pi_{\text{dcc}}). \tag{20}$$

It is now evident that $\mathcal{S}_0(\pi_{\text{dcc}})$ is a monotonically increasing function of $m(C_q)$ and lower-bounds $\mathcal{S}(\pi_{\text{dcc}})$, being equal if and only if $q_1 = q_2$.

Consider, on the other hand, that the EOF $\mathcal{E}(\pi_{\text{dcc}})$ of a pure de-cross-correlated state is also a monotonically increasing function of $m(C_q)$[6,7], given by

$$\mathcal{E}(\pi_{\text{dcc}}) = h\left[\sqrt{m(C_q)} - \sqrt{m(C_q) - 1}\right], \tag{21}$$

where

$$h[x] := \frac{(1+x)^2}{4x}\ln\left(\frac{(1+x)^2}{4x}\right) - \frac{(1-x)^2}{4x}\ln\left(\frac{(1-x)^2}{4x}\right). \tag{22}$$

and we also define $h_0[x] := h[e^{-x}]$. It then follows from Eqs. (20) and (21) that

$$\mathcal{E}(\pi_{\text{dcc}}) = h\left[e^{\ln\left(\sqrt{m(C_q)} - \sqrt{m(C_q)-1}\right)}\right] = h\left[e^{-\ln\left(\sqrt{m(C_q)} + \sqrt{m(C_q)-1}\right)}\right] = h_0\left[\mathcal{S}_0(\pi_{\text{dcc}})\right] \leq h_0\left[\mathcal{S}(\pi_{\text{dcc}})\right] \tag{23}$$

for all pure de-cross-correlated states, with equality holding if and only if $q_1 = q_2$. The $q_1 = q_2$ condition physically corresponds to the state from a balanced beam splitter mixing two non-correlated modes, which results in a state with two identical modes. Combining $\mathcal{E}(\pi_{\text{dcc}}) = h_0[\mathcal{S}(\pi_{\text{dcc}})] \iff q_1 = q_2$ with $\mathcal{E}(\pi_{\text{dcc}}) \leq \mathcal{P}(\pi_{\text{dcc}}) \leq h_0[\mathcal{S}(\pi_{\text{dcc}})]$, we conclude that a pure de-cross-correlated state can satisfy $\mathcal{E}(\pi_{\text{dcc}}) = \mathcal{P}(\pi_{\text{dcc}})$ if and only if $q_1 = q_2$. We also conclude that this special class of de-cross-correlated states satisfy $\mathcal{P}(\pi_{\text{dcc}}) = h_0[\mathcal{S}(\pi_{\text{dcc}})]$ and thus saturate the upper bound in Eq. (14).

The above discussion suggests a way to maximise a given state's EOF via passive operations. For mixed states, both the EOF and the SOF are defined via a convex optimisation over all possible pure state decompositions, so a mixed state σ could saturate the upper bound in Eq. (14) if its potential-saturating pure state π_{opt} is de-cross-correlated (i.e., of the form in Eq. (16)) and has $q_1 = q_2$. This is the fundamental idea behind our algorithm, which first performs passive operations required to decouple the squeezing between two modes of a given input state, then de-cross-correlates this state passively, and finally, by appropriately mixing with an ancillary vacuum, removes any excess noise in order to maximise the EOF.

References

1. Arvind, Dutta, B., Mukunda, N. & Simon, R. The real symplectic groups in quantum mechanics and optics. *Pramana* **45**, 471–497, DOI: 10.1007/BF02848172 (1995).
2. Braunstein, S. L. Squeezing as an irreducible resource. *Phys. Rev. A* **71**, 055801, DOI: 10.1103/PhysRevA.71.055801 (2005).
3. Assad, S. M., Gu, M., Li, X. & Lam, P. K. Decoupling cross-quadrature correlations using passive operations. *Phys. Rev. A* **102**, 022615, DOI: 10.1103/PhysRevA.102.022615 (2020).
4. Tserkis, S. *et al.* Maximum entanglement of formation for a two-mode Gaussian state over passive operations. *Phys. Rev. A* **102**, 052418, DOI: 10.1103/PhysRevA.102.052418 (2020).
5. Duan, L.-M., Giedke, G., Cirac, J. I. & Zoller, P. Inseparability criterion for continuous variable systems. *Phys. Rev. Lett.* **84**, 2722–2725, DOI: 10.1103/PhysRevLett.84.2722 (2000).
6. Adesso, G. & Illuminati, F. Gaussian measures of entanglement versus negativities: Ordering of two-mode Gaussian states. *Phys. Rev. A* **72**, 032334, DOI: 10.1103/PhysRevA.72.032334 (2005).
7. Wolf, M. M., Giedke, G., Krüger, O., Werner, R. F. & Cirac, J. I. Gaussian entanglement of formation. *Phys. Rev. A* **69**, 052320, DOI: 10.1103/PhysRevA.69.052320 (2004).
8. Idel, M., Lercher, D. & Wolf, M. M. An operational measure for squeezing. *J. Phys. A: Math. Theor.* **49**, 445304, DOI: 10.1088/1751-8113/49/44/445304 (2016).
